# Cognitive function in recovered COVID-19 Lebanese patients with schizophrenia

**DOI:** 10.1186/s12991-023-00435-4

**Published:** 2023-03-11

**Authors:** Chadia Haddad, Angela Chamoun, Hala Sacre, Souheil Hallit, Pascale Salameh, Benjamin Calvet

**Affiliations:** 1 Research Department, Psychiatric Hospital of the Cross, P.O. Box 60096, Jal Eddib, Lebanon; 2 Inserm U1094, IRD UMR270, Univ. Limoges, CHU Limoges, EpiMaCT - Epidemiology of chronic diseases in tropical zone, Institute of Epidemiology and Tropical Neurology, OmegaHealth, Limoges, France; 3 Institut National de Santé Publique, d’Épidémiologie Clinique et de Toxicologie-Liban (INSPECT-LB), Beirut, Lebanon; 4 School of Health Sciences, Modern University for Business and Science, Beirut, Lebanon; 5 School of Medicine, Lebanese American University, Byblos, Lebanon; 6 Faculty of Sciences, Lebanese University, Fanar, Lebanon; 7 School of Medicine and Medical Sciences, Holy Spirit University of Kaslik, P.O. Box 446, Jounieh, Lebanon; 8 Applied Science Research Center, Applied Science Private University, Amman, Jordan; 9 Department of Primary Care and Population Health, University of Nicosia Medical School, 2417 Nicosia, Cyprus; 10 Faculty of Pharmacy, Lebanese University, Beirut, Lebanon; 11 Centre Hospitalier Esquirol, Pôle Universitaire de Psychiatrie de l’Adulte et de la Personne Âgée, d’Addictologie, Centre Mémoire de Ressources et de Recherche, 87000 Limoges, France; 12 Centre Hospitalier Esquirol, Unité de Recherche et d’Innovation, 87000 Limoges, France

**Keywords:** Cognition, COVID-19, Schizophrenia, Infection, Disease

## Abstract

**Introduction:**

It remains unclear whether COVID-19 which is an infectious disease caused by the SARS-CoV-2 virus is associated with the deterioration of cognitive function among patients with schizophrenia. This study aimed to evaluate changes in cognitive function before and after COVID-19 and associated factors among patients with schizophrenia at the Psychiatric Hospital of the Cross (HPC).

**Methods:**

A prospective cohort study was conducted among 95 patients with schizophrenia followed from mid-2019 until June 2021 at the Psychiatric Hospital of the Cross (HPC). This cohort was divided into a group diagnosed with COVID-19 (*n* = 71) and another not diagnosed with COVID-19 (*n* = 24). The questionnaire included the Brief Assessment of Cognition in Schizophrenia (BACS), Positive and Negative Syndrome Scale (PANSS), Calgary Depression Scale for Schizophrenia (CDSS), and Activities of Daily Living (ADL).

**Results:**

The repeated-measures ANOVA showed no significant effect of time and the interaction between time and being diagnosed or not with COVID-19 on cognition. However, being diagnosed or not with COVID-19 had a significant effect on global cognitive function (*p* = 0.046), verbal memory (*p* = 0.046), and working memory (*p* = 0.047). The interaction between being diagnosed with COVID-19 and cognitive impairment at baseline was significantly associated with a higher cognitive deficit (Beta = 0.81; *p* = 0.005). Clinical symptoms, autonomy, and depression were not associated with the cognition (*p* > 0.05 for all).

**Conclusion:**

COVID-19 disease affected global cognition and memory: patients diagnosed with COVID-19 had more deficits in these domains than those without COVID-19. Further studies are necessary to clarify the variation of cognitive function among schizophrenic patients with COVID-19.

## Introduction

Various neurological manifestations are associated with COVID-19 [1–3]. They may involve the central nervous system causing headache, dizziness, impaired consciousness, acute cerebrovascular disease, and epilepsy, or the peripheral nervous system contributing to a loss of smell, a loss of taste, and Guillain–Barre syndrome, as well as skeletal muscles pain [4]. Neuropsychiatric manifestations may also occur, including encephalopathy, cognitive impairment, mood swings, insomnia, suicide, and psychosis [2].

The mechanism and specific pathophysiological pathways through which COVID-19 causes neurological, psychiatric, and cognitive dysfunctions are yet unknown but are likely multifactorial [5]. The effects of SARS-CoV-2 on the brain could be indirect (mediated by oxygen starvation of the brain and/or by the body’s extreme inflammatory response in severely affected patients), direct (mediated by virus invasion in the brain), or both [6, 7].

Post-COVID-19 cognitive impairment may represent an important clinical feature of the disease and impose an added and long-lasting burden. New findings suggest that a significant number of patients face long-term cognitive impairments such as memory problems and an incapability to concentrate, even after they have recovered from COVID-19 [8, 9]. A recent cohort study on 57 patients recovering from COVID-19 showed changes in working memory, set-shifting, divided attention, and processing speed. Another study showed that people who had recovered from COVID-19, including those no longer reporting symptoms, presented significant cognitive deficits compared to controls [8]. Some findings indicated that hippocampus exposure to SARS-CoV-2 may lead to a potential cognitive dysfunction in patients with COVID-19, including a decline in memory, attention, and concentration [10], which might accelerate the onset of neurodegenerative disorders such as Alzheimer’s disease in the long term [10]. A cohort study evaluating the immediate effects of SARS-CoV-2 infection two to three weeks after recovery among 29 patients indicated a potential cognitive dysfunction in the attention area in the studied population [11]. Another study among 29 COVID-19 patients at the Bispebjerg Hospital in Denmark reported a significant cognitive impairment four months after discharge [12]. The most impaired cognitive domains were verbal learning, working memory, and executive functions, with some patients experiencing severe cognitive difficulties in daily life [12]. One more cohort study done among 1733 patients at Jin Yin-tan Hospital in Wuhan found that patients who had recovered from COVID-19 would face fatigue or muscle weakness and sleep difficulties 6 months after recovery. The risk of anxiety or depression was higher in more severely ill cases [13].

The COVID-19 pandemic has a high impact on populations, particularly vulnerable ones, such as patients with schizophrenia. A recent systematic review and meta-analysis of 16 observational studies in seven countries showed that mental health disorders were associated with increased COVID-19-related mortality [14]. Moreover, *Toxoplasma gondii* infections were reportedly linked to higher mortality in COVID-19 patients with schizophrenia, either directly or indirectly [15]. Also, schizophrenia patients are at greater risk of transmitting the virus because they find it hard to apply the protective measures recommended to prevent the infection due to psychotic symptoms and an immune-related mechanism [16]. Cognitive impairment is considered a core feature of schizophrenia that appears at the onset of the illness and might be secondary to drug effects [17]. In schizophrenia patients, several cognitive functions are impaired, including memory, attention, motor skills, executive function, and social cognition [18]. Antipsychotic treatments used to improve psychotic symptoms and cognitive function might, in turn, contribute to the cognitive impairment seen in many patients with schizophrenia [19]. Effective symptoms’ management with antipsychotics may have a protective effect against COVID-19, a theory that is consistent with the pathophysiology of COVID-19 [20]. A retrospective study among 698 individuals with severe mental disorders (SMD) showed that individuals on antipsychotic treatment had a lower risk of SARS-CoV-2 infection and had a likely better disease prognosis [20].

In Lebanon, the Psychiatric Hospital of the Cross (HPC), the largest inpatient mental institution, has reacted quickly to the COVID-19 pandemic in the country by endorsing containment actions, such as temporarily suspending admissions and limiting family visits. Despite these measures, there were two COVID-19 outbreaks among patients at the HPC, one in September 2020 and another in January 2021, with 94 and 316 cases identified, respectively. The first COVID-19 case was detected in a registered nurse; the virus quickly spread to most hospitalized patients, infecting 410 patients (76.51% of all patients).

To the best of our knowledge, no study has yet explored the consequences of COVID-19 on cognition in patients diagnosed with schizophrenia, and it remains unknown whether the infection is associated with the deterioration of cognitive function among patients with schizophrenia. It is also unclear how many patients keep on showing clinically relevant cognitive impairments after their recovery. Therefore, this prospective cohort study aimed to evaluate the changes in cognitive function before and after COVID-19 and associated factors among patients with schizophrenia at the Psychiatric Hospital of the Cross (HPC).

## Material and methods

### Study design and participants

A prospective cohort study was conducted among 95 patients with schizophrenia residing in the Psychiatric Hospital of the Cross (HPC) and followed from mid-2019 until June 2021. The HPC is the largest psychiatric care hospital in Lebanon and the Middle East, with more than 887 psychiatric beds for a total of 1301 admissions per year. More than 400 psychiatric patients have been residing in the hospital for more than ten years. There are no financial/ethnic group restrictions for admission. Our sample was divided into a group diagnosed with COVID-19 (*n* = 71) and another not diagnosed with COVID-19 (*n* = 24). All the patients selected in this study had undergone cognitive tests between June and December 2019 and in June and July 2021. The mean duration between the two assessments was 20.65 ± 1.47 months. Patients were followed for approximately 6.62 ± 1.94 months after the spread of COVID-19 in the hospital. Inclusion criteria were schizophrenic patients diagnosed or not with COVID-19, aged 18 to 60, who had previously undergone a cognitive test, completed at least five years of education, and fulfilled the DSM-5 criteria for schizophrenia. A confirmed case of COVID-19 was defined as the positive real-time reverse transcriptase-polymerase chain reaction (RT-PCR) assay result of nasopharyngeal swab specimens. Exclusion criteria were conditions that would influence cognitive performance, i.e., brain trauma, neurological disorder, or current substance use disorder.

### Measures

The same methodology was described in a previous study [21]. The questionnaire used was in Arabic, the native language in Lebanon. The first section assessed the sociodemographic and clinical characteristics of participants, including age, gender, education level, marital status, monthly salary, family history of mental illnesses, hospitalization length, illness duration, and the number of hospitalizations. COVID-19 was categorized into asymptomatic, mild, moderate, severe, and critical, as per the WHO classification [22]. Persistent post-COVID-19 symptoms were also recorded using a checklist including: cough, fever, dyspnea, chest pain, diarrhea, abdominal pain, loss of taste, loss of smell, sore throat, nausea, vomiting, headache, myalgia, arthralgia, fatigue and rash. The medications that patients were taking were retrieved from their medical records at the time of data collection. For antipsychotics, the chlorpromazine dose equivalent was calculated using the Andreasen method [23]. The Anticholinergic Drug Scale (ADS) developed by Carnahan et al. was used to rank anticholinergic medications, where each medication was assigned a numerical value [24]. The total ADS scores were calculated by summing the values of all scheduled medications used by each participant.

The second section of the questionnaire included the following measures:

#### The Brief Assessment of Cognition in Schizophrenia (BACS)

The BACS, recently validated in Lebanon [25] is a neuropsychological battery used to evaluate cognitive functioning in patients with schizophrenia [26]. It consists of six subscales: list learning (using the 15 items) (verbal memory), digit sequencing (working memory), token motor task (psychomotor function), semantic fluency (verbal fluency), symbol coding (attention and speed of information processing), and Tower of London (executive function) [26]. In this study, the BACS was administered with the standard strategies using the same version before and after the COVID-19 infection.

#### The Positive and Negative Syndrome Scale (PANSS)

The PANSS, validated in Lebanon [27], is a 30-item questionnaire organized into three subscales: positive symptoms (7 items), negative symptoms (7 items), and general psychopathology (16 items) [28]. All items are scored from 1 (absence of symptoms) to 7 (extremely severe symptoms) [28]. The total score was calculated by summing all answers, with higher scores indicating more severe symptoms [28].

#### Calgary Depression Scale for Schizophrenia (CDSS)

The CDSS is a 9-item structured interview scale developed by Addington et al. [29] to assess depression in patients with schizophrenia. The Arabic version of the CDSS was validated among 204 subjects recruited from the Arab population residing in Doha, Qatar (102 people living with schizophrenia and 102 controls subjects) [30]. Eight structured questions assess depression, hopelessness, self-depreciation, guilty ideas of reference, pathological guilt, morning depression, early wakening, and suicide, followed by one observation item (observed depression). Higher scores represent a greater level of depression [29].

#### Activities of Daily Living (ADL)

The ADL is a 6-item scale that assesses overall functional activity in: (1) bathing, (2) dressing, (3) going to the toilet, (4) transferring (movement), (5) continence, and (6) feeding [31]. Nasser and Doumit had validated the Arabic version of the scale in a sample of Lebanese elderly living in nursing homes [32]. In the Arabic version, the six components are scored 0, 0.5, or 1. The total ADL score ranges from 0 to 6, where 6 entails complete independence and 0 complete dependence. The total mean score was calculated by summing the scores of the six items, where a higher score indicates a greater autonomy level.

### Statistical analysis

The Statistical Package for the Social Sciences (SPSS) version 25 was used for data entry and analysis. A descriptive analysis was performed, where categorical variables were expressed as absolute frequencies and percentages and quantitative variables as means and standard deviations. The sample was normally distributed as verified by the skewness and kurtosis of the BACS score (z below |1.96|) [33].

The composite standardized z-score of the BACS was calculated by averaging the total score from the mean total score of the BACS of a healthy control group selected from the same database from a previous study [21, 22]. In addition, a normative cognitive change was calculated based on the group of patients without COVID-19 to explore the proportion of patients whose cognition deteriorated compared with this normative cognitive change. Among patients without COVID-19, the formula was *X*_2_ − *X*_1_/SD, where *X*_1_ = the mean BACS composite standardized score before COVID-19, *X*_2_ = the BACS composite standardized score after COVID-19, and SD = pre-COVID-19 period standard deviation of the BACS measure. The result of the variation was −0.74, considered the cut-off value, where a BACS composite standardized score lower than −0.74 after COVID-19 reflected cognitive deterioration and values closer to zero indicated cognitive improvement.

A repeated-measures ANOVA was conducted to evaluate the variation in time of BACS total composite standardized score and subscores between patients infected or not with COVID-19 adjusted for age, gender, education level, duration between BACS assessment, chlorpromazine equivalent dose, and ADS score.

A multivariate analysis was performed using the GLM Univariate analysis and taking the global cognition after COVID-19 infection as the dependent variable. The fixed factor was being diagnosed or not with COVID-19, adjusted for age, gender, education level, clinical symptoms, depression, cognition at baseline, duration between cognitive assessment, chlorpromazine equivalent dose, autonomy, and ADS score. A value of *p* < 0.05 was considered significant. The internal consistency of the scales was assessed using Cronbach’s alpha.

## Results

### Sociodemographic characteristics

Our cohort consisted of 95 patients divided into a group of patients diagnosed with COVID-19 (*n* = 71) and another not diagnosed with COVID-19 (*n* = 24). Table 1 summarizes the sociodemographic characteristics of the participants. The mean age of patients diagnosed with schizophrenia was 49.86 ± 7.36 years, with 62.1% males. Most participants (83.2%) were single, had a low monthly income (below < 1000$) (53.7%), and a secondary level of education (54.7%). Also, 37.2% had a family history of psychiatric disease. The mean lengths of illness and hospitalization were 22.49 ± 9.05 and 13.74 ± 8.32 years, respectively. The mean number of hospitalizations was 6.08 ± 4.90 times.

**Table 1:** Sociodemographic characteristics of the studied sample (*N* = 95)

	Frequency (%)
Gender	
Male	59 (62.1%)
Female	36 (37.9%)
Education level	
Complementary	28 (29.5%)
Secondary	52 (54.7%)
University	15 (15.8%)
Marital status	
Single	79 (83.2%)
Married	5 (5.3%)
Divorced	10 (10.5%)
Widowed	1 (1.1%)
Monthly income	
No income	24 (25.3%)
< 1000 $	51 (53.7%)
1000–2000 $	20 (21.1%)
> 2000 $	0 (0%)
Family history of psychiatric illness	
Yes	35 (37.2%)
No	59 (62.8%)
	Mean ± SD
Duration of hospitalization in years	13.74 ± 8.32
Duration of illness in years	22.49 ± 9.05
Number of hospitalizations	6.08 ± 4.90
Age in years	49.86 ± 7.36

### Description of COVID-19 characteristics

Of the total sample, 74.7% have been diagnosed with COVID-19, and almost all (93.0%) had mild symptoms or were asymptomatic. Only 22.9% had more than two symptoms post-COVID-19. The mean duration between SARS-CoV-2 infection and cognitive assessment was 6.62 ± 1.94 months (Table 2).

**Table 2:** Description of COVID-19 characteristics among patients with schizophrenia

	Frequency (%)
Patients diagnosed with COVID-19	
Yes	71 (74.7%)
No	24 (25.3%)
Severity of the COVID-19 infection	
Asymptomatic	32 (45.1%)
Mild	34 (47.9%)
Moderate	0 (0.0%)
Severe	5 (7.0%)
Presence of post-COVID-19 symptoms	
Cough	4 (5.7%)
Fever	2 (2.9%)
Dyspnea	14 (20.0%)
Chest pain	9 (12.9%)
Diarrhea	4 (5.7%)
Abdominal pain	6 (8.6%)
Loss of taste	5 (7.1%)
Loss of smell	5 (7.1%)
Sore throat	3 (4.3%)
Nausea	8 (11.4%)
Vomiting	3 (4.3%)
Headache	9 (12.9%)
Myalgia	7 (10.0%)
Arthralgia	13 (18.6%)
Fatigue	37 (52.9%)
Rash	0 (0%)
Number of post-COVID-19 symptoms	
0	26 (37.1%)
1	16 (22.9%)
2	12 (17.1%)
> 2	16 (22.9%)
	Mean ± SD
Duration between the cognitive evaluation at baseline and post-COVID period cognitive assessment by month	20.65 ± 1.47
Duration between the infection by the SARS-CoV-2 virus and post cognitive assessment by month	6.62 ± 1.94

### Reliability of the scales used in this study

At baseline, Cronbach’s alpha values of the scales used in this study were 0.877 for the BACS scale, 0.787 for the positive PANSS scale, 0.788 for the negative PANSS scale, 0.839 for the general psychopathology PANSS scale, 0.877 for the total PANSS scale, 0.825 for the Calgary scale, and 0.738 for the ADL scale.

After the COVID-19 infection, these values were 0.840 for the BACS scale, 0.400 for the positive PANSS scale, 0.648 for the negative PANSS scale, 0.685 for the general psychopathology PANSS scale, 0.816 for the Calgary scale, and 0.702 for the ADL scale.

### Variation of cognition before and after the COVID-19 infection

Table 3 presents the variation in cognitive function of all patients (whether or not infected with SARS-CoV-2) before and after the COVID-19 outbreak in the hospital. A normative cognitive change was calculated based on the variation of the BACS composite score among those who were not infected with SARS-CoV-2. Of those who had COVID-19, 68 (95.8%) had a major cognitive deterioration. Before the COVID-19 period, a significant difference was found in the BACS total composite score between patients who had a major cognitive deterioration and those who did not [*M*_*BACS among deteriorated group*_ = −3.01 vs. *M*_*BACS among non-deteriorated group*_ = −0.48, *p* < 0.001]. After the COVID-19 period, a significantly higher cognitive deficit was found among patients with major cognitive deterioration compared to those without [*M*_*BACS among deteriorated group*_ = −3.30 vs. *M*_*BACS among non-deteriorated group*_ = 0.34, *p* < 0.001].

**Table 3:** Repeated-measures ANOVA of the cognitive function before and after COVID-19 infection in patients with schizophrenia

	Patients having COVID-19 (*n* = 71)				Patients without COVID-19 (*n* = 24)				Repeated-measures ANOVA					
									Time effect		Group (COVID-19 positive vs negative) effect		Interaction (group *x* Time)	
	Before COVID-19 period	After COVID-19 period	*t*	*p*–value	Before COVID-19 period	After COVID-19 period	*t*	*p*-value	*p*	Effect size (*n*^2^)	*p*	Effect size (*n*^2^)	*p*	Effect size (*n*^2^)
	Mean ± SD [95% CI]	Mean ± SD [95% CI]			Mean ± SD [95% CI]	Mean ± SD [95% CI]								
Cognitive function														
BACS (global score)	−2.93 ± 1.25 [−3.23;−2.63]	−3.20 ± 1.37 [−3.52;−2.87]	2.70	0.009	−2.91 ± 1.20 [−3.42;−2.41]	−3.16 ± 1.04 [−3.60; −2.72]	1.16	0.256	0.749	0.001	0.046	3.59	0.409	0.008
Verbal memory (List learning)	−2.22 ± 1.05 [−2.47;−1.97]	−1.57 ± 1.55 [−1.93;−1.20]	−4.18	< 0.001	−2.06 ± 0.96 [−2.47;−1.65]	−1.59 ± 1.15 [−2.08;−1.10]	−2.19	0.039	0.416	0.008	0.046	3.55	0.920	0.001
Working memory (Digit sequencing)	−1.86 ± 1.26 [−2.16;−1.56]	−2.43 ± 1.60 [−2.81;−2.05]	4.55	< 0.001	−1.97 ± 1.23 [−2.49;−1.45]	−2.11 ± 1.40 [−2.70;−1.52]	0.54	0.592	0.051	0.046	0.047	3.53	0.081	0.037
Motor speed (Token motor task)	−2.46 ± 1.11 [−2.72;−2.20]	−3.21 ± 1.20 [−3.50;−2.92]	4.99	< 0.001	−2.58 ± 1.03 [−3.01;−2.14]	−3.28 ± 1.07 [−3.73;−2.82]	2.66	0.014	0.958	0.001	0.301	1.94	0.385	0.009
Verbal fluency (Semantic, alphabetical)	−1.56 ± 0.97 [−1.79;−1.33]	−1.49 ± 1.07 [−1.74;−1.23]	−1.05	0.298	−1.44 ± 0.89 [−1.82;−1.06]	−1.45 ± 0.81 [−1.80;−1.11]	0.08	0.935	0.341	0.011	0.093	3.03	0.682	0.002
Attention and speed of information processing (Symbol coding)	−2.39 ± 1.27 [−2.69;−2.09]	−2.61 ± 0.99 [−2.85;−2.38]	2.68	0.009	−2.43 ± 0.76 [−2.76;−2.11]	−2.57 ± 0.85 [−2.93;−2.21]	0.79	0.435	0.694	0.002	0.149	2.61	0.548	0.004
Executive function (Tower of London)	−2.28 ± 2.07 [−2.77;−1.79]	−2.61 ± 2.00 [−3.08;−2.14]	1.64	0.106	−2.08 ± 1.89 [−2.88;−1.28]	−2.46 ± 1.99 [−3.30;−1.62]	1.47	0.155	0.111	0.031	0.080	3.07	0.570	0.004

The repeated-measures ANOVA was performed taking the group (COVID + vs COVID-) by time adjusted for age, gender, education level, duration between BACS assessment, chlorpromazine equivalent dose and ADS score

**Fig. 1:**
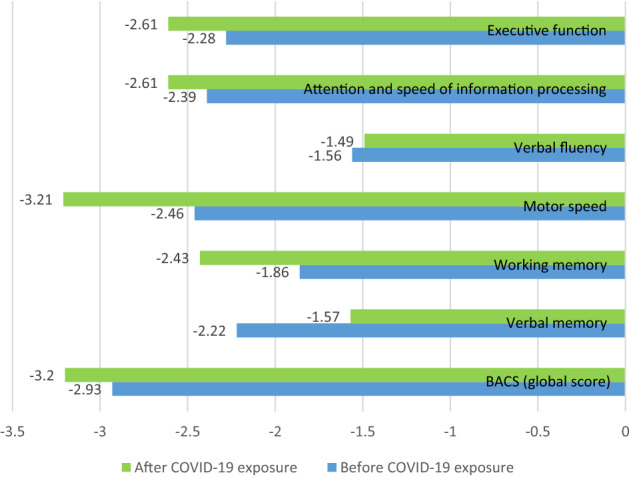
Variation of the measures used before and after COVID-19 infection among patients infected with COVID-19

A paired-sample *t*-test was conducted to compare the cognitive function before and after the SARS-CoV-2 infection, independent of time. The results showed that, among patients diagnosed with COVID-19, the global cognitive function [*M*_*BACS baseline*_ = -2.93 vs. *M*_*BACS follow-up*_ = -3.20, *p* = 0.009], working memory [*M*_*BACS baseline*_ = −1.86 vs. *M*_*BACS follow-up*_ = −2.43, *p* < 0.001], motor speed [*M*_*BACS baseline*_ = −2.46 vs. *M*_*BACS follow-up*_ = −3.21, *p* < 0.001], and attention and speed of information processing [*M*_*BACS baseline*_ = −2.39 vs. *M*_*BACS follow-up*_ = −2.61, *p* = 0.009] have decreased (deteriorated) after the COVID-19 period, contrary to verbal memory that improved (ameliorated) after the COVID-19 period [*M*_*BACS baseline*_ = −2.22 vs. *M *_*BACS follow-up*_ = −1.57, *p* < 0.001] (Fig. 1).

Among patients without COVID-19, the results showed that the motor speed has decreased after the COVID-19 period [*M*_*BACS baseline*_ = −2.58 vs. *M*_*BACS follow-up*_ = −3.28, *p* = 0.014]. However, the verbal memory has improved after the COVID-19 period *[M*_*BACS baseline*_ = −2.06 vs. *M*_*BACS follow-up*_ = −1.59, *p* = 0.039].

After that, a repeated-measures ANOVA was performed to show the effect of time, group (being diagnosed or not with COVID-19), and the interaction of time and group on cognition. The results showed no significant effect of time on global cognition [*F* (1, 81) = 0.103, *p* = 0.749] and the interaction between time and being diagnosed or not with COVID-19 was not significant [*F* (1, 81) = 0.688, *p* = 0.409]. Also, no significant effect of time and interaction of time with the group (being diagnosed or not with COVID-19) was found for all the cognition subtests [*p* > 0.05 for all]. However, a significant effect of group was found for the global cognitive function [*F* (1, 81) = 4.10, *p* = 0.046], verbal memory [*F* (1, 81) = 4.11, *p* = 0.046], and working memory [*F* (1, 81) = 4.07, *p* = 0.047]. Higher means of global cognitive deficit [*M*_*Total BACS COVID*+_  = −3.20 vs *M*_*Total BACS COVID-*_ = −2.63], verbal memory deficit [*M*_*verbal memory in COVID*+_  =  * 1.98 vs *M*_*verbal memory in COVID-*_ = −1.43], and working memory deficit [*M*_*working memory in COVID*+_  = −2.28 vs *M*_*working memory in COVID-*_ = −1.64] were significantly found among patients who were COVID-19 positive compared to those who were not.

A significant decline in clinical symptoms (positive, negative, and general psychopathology PANSS scores) was found among patients diagnosed with COVID-19 after the COVID-19 period. Patients without COVID-19 had a decline in clinical symptoms (negative and general psychopathology PANSS scores). No significant change was found for depression, chlorpromazine equivalent dose, and ADS scores before and after COVID-19 among the two groups of patients (Table 4).

**Table 4:** Variation of the measures used before and after COVID-19 period in patients with schizophrenia

	Patients having COVID-19 (*n* = 71)			Patients without COVID-19 (*n* = 24)		
	Before COVID-19 period	After COVID-19 period	*P* -value	Before COVID-19 period	After COVID-19 period	*p*-value
	Mean ± SD	Mean ± SD		Mean ± SD	Mean ± SD	
**Clinical symptoms**						
Positive PANSS	21.73 ± 10.25	17.38 ± 6.31	**0.001**	18.62 ± 8.54	17.37 ± 6.76	0.467
Negative PANSS	17.29 ± 8.04	13.63 ± 7.09	**0.004**	18.25 ± 7.98	13.70 ± 6.59	**0.022**
General psychopathology PANSS	47.63 ± 16.38	34.91 ± 11.10	** < 0.001**	45.00 ± 18.78	33.04 ± 11.33	**0.002**
**Calgary scale (depression)**	4.88 ± 4.86	4.70 ± 4.76	0.748	5.04 ± 4.44	4.58 ± 4.51	0.665
**ADL**	5.66 ± 0.79	5.63 ± 0.86	0.817	5.85 ± 0.54	5.83 ± 0.43	0.714
**Chlorpromazine equivalent dose**	1069.21 ± 1178.24	1070.38 ± 1034.34	0.990	1622.00 ± 1208.56	1830.95 ± 1114.72	0.051
**ADS score**	7.26 ± 3.08	7.19 ± 2.99	0.753	8.37 ± 3.49	8.00 ± 3.63	0.205

Values marked in bold are significant

### Multivariable analysis

The results of multivariable analysis, taking the global cognition after the COVID-19 infection as the dependent variable, showed that having a secondary level of education (Beta = 0.52) and the interaction between being diagnosed with COVID-19 and cognitive impairment at baseline (Beta = 0.81) were significantly associated with higher cognitive deficit.

Clinical symptoms, autonomy, and depression were not associated with cognition (*p* > 0.05 for all) (Table 5).

**Table 5:** Multivariable analysis taking the global cognition after the COVID-19 period as the dependent variable

	Beta	Effect size (*n*^2^)	*p*-value	Confidence interval	
				Lower	Upper
COVID-19 infection (Yes vs No)	−3.783	0.006	0.540	−16.043	8.478
Gender (Female vs Male)	0.110	0.002	0.737	−0.541	0.760
Education level (University vs complementary)	0.402	0.022	0.235	−0.268	1.072
Education level (Secondary vs complementary)	0.521	0.063	0.043	0.017	1.025
Positive PANSS	−0.018	0.004	0.601	−0.086	0.050
Negative PANSS	−0.011	0.002	0.720	−0.072	0.050
General psychopathology PANSS	−0.003	0.000	0.859	−0.031	0.026
Depression (Calgary scale)	0.021	0.001	0.775	−0.125	0.167
BACS composite score at baseline	0.027	0.000	0.920	−0.511	0.566
duration between BACS assessment	−0.121	0.007	0.516	−0.492	0.249
chlorpromazine equivalent dose	−0.003	0.018	0.278	−0.001	0.000
ADS score	−0.082	0.031	0.159	−0.196	0.033
Age	−0.044	0.033	0.147	−0.103	0.016
Autonomy (total ADL)	−0.004	0.000	0.991	−0.840	0.831
Interaction being diagnosed with COVID-19 and cognition at baseline	0.816	0.116	0.005	0.255	1.377

Variables entered in the model: being diagnosed or not with COVID-19, age, gender, education level, clinical symptoms, depression, cognition at baseline, duration between cognitive assessment, chlorpromazine equivalent dose, autonomy and ADS score

## Discussion

To our knowledge, this study is the first to evaluate the variation of objective cognitive function among patients with schizophrenia, diagnosed or not with COVID-19. The main findings in this study were that patients with schizophrenia infected with SARS-CoV-2 exhibited a major cognitive deterioration on the BACS total score and, more specifically, on the working memory, motor speed and attention, and speed of information processing domains. Also, SARS-CoV-2 infection affected global cognition in all patients, verbal memory, and working memory, where patients diagnosed with COVID-19 had more deficits in these domains than those without COVID-19. These results are consistent with those of other studies showing that patients with viral infections had cognitive impairment [34–36]. In our study, the variation of impairment in cognitive domains could be explained by the differences in methods between studies, such as the cognitive scales used and the population studied.

Experimentally, Zika virus infections profoundly impact neurodevelopmental outcomes, including cognitive impairment, in the long term [37]. In patients with acute respiratory distress syndrome (ARDS), cognitive impairment is common; studies have found that 70 to 100% of ARDS patients are cognitively impaired when they leave the hospital, 46–80% one year later, and 20% after five years [38, 39]. A recent study reported cognitive impairment, specifically in the attention domains, among 29 patients who recovered from COVID-19 [11]. Another study in Denmark among 29 COVID-19 patients admitted to the hospital found that cognitive impairment ranged from 59 to 65% 3–4 months after their discharge, with verbal learning and executive functions being the most impaired domains [12]. Other findings show that 28–56% of patients with mild or asymptomatic COVID-19 had cognitive impairment, mainly attention and executive dysfunctions [40]. Viral infections-related diseases such as COVID-19, which invade the central nervous system and affect the cardiopulmonary system, have been linked to neurologic complications, delayed neurodevelopment, and decreased cognitive performance [41]. However, longitudinal studies should be performed among patients with schizophrenia to assess the long-term impact of SARS-CoV-2 infection on cognitive function.

In this study, the most impaired cognitive domains were working memory, motor speed, and attention among COVID-19 patients. A recent systematic review, including six studies conducted among recovered COVID-19 patients, has demonstrated an increase in inflammatory markers known to have a profound impact on working memory and attention [42]. A retrospective meta-analysis of SARS-CoV and MERS-CoV outbreaks had reported similar results, where the most common impaired cognition was concentration, attention, and memory in recovered patients [43]. A cross-sectional study among 18 patients who recovered from mild-to-moderate COVID-19 has found that short-term memory, attention, and concentration were particularly affected by COVID-19 [10]. Another study among 29 patients who recovered from COVID-19 has reported that attention was the most impaired cognitive domain [11]. COVID-19 showed to induce brain damage by direct infection, strokes, and oxygen deprivation [44]. The tentative mechanism of coronavirus is the evasion adopted by the virus with other factors, such as the viral load or the impaired immune response that can contribute to the immunopathogenesis of COVID-19 [45]. Also, the inflammatory process caused by the coronavirus and the resulting cytokine storm can contribute to neurological changes and destroy the neuronal connection, thus deteriorating memory, attention, and executive function [44]. A high number of patients with COVID-19 may develop long-term symptoms, which profile and timeline remain unknown [46]. Those patients with prolonged illness are identified as “Long COVID” [46].

Our results also showed that the motor task has deteriorated in the whole sample. The decline in motor tasks among patients with COVID-19 could be explained by the schizophrenia illness itself, as the movements of these patients could be extremely slowed, and psychomotor activity is sometimes decreased due to the disease, although neuroleptic treatment appears to affect some aspects of psychomotor performance [47]. Several studies conducted among patients with schizophrenia have shown that the motor task was the most impaired cognitive function [25, 48, 49].

Furthermore, contrary to previous findings [10, 50, 51], our study showed an improvement in verbal memory after the COVID-19 period in both groups, likely due to treatment with antipsychotic medications [52] or remediation treatment [53] rather than infection. In both groups, psychotic symptoms, including negative symptoms, were improved, which might have ameliorated the verbal memory. Future studies are necessary to understand the factors influencing verbal memory among chronic patients with schizophrenia.

Our study showed that clinical symptoms have improved after the COVID-19 period in both groups. However, positive symptoms have improved only in patients infected with the virus. A possible explanation is that psychotic symptoms responded well to medications and decreased over the course of the illness. The low depression rates detected among patients did not differ after the COVID-19 period, assuming that the patients did not experience depressive thoughts or any psychological reaction due to the virus. Also, it could be that patients living in a community do not face the stress induced by COVID-19 nor the anxiety of catching the virus. Previous studies have indicated important links between anxiety and depression and viral diseases, such as influenza A (H1N1) and other influenza viruses, varicella-zoster virus, herpes simplex virus, HIV/AIDS, and hepatitis C [54]. Our results also showed that activity of daily living was good among patients and remained the same before and after the COVID-19 period. A possible explanation of these results could be that people with schizophrenia residing in clinical settings have limited occupational performance and community functioning and spend their time doing sedentary activities [55].

The use of antipsychotic medications did not change before and after the COVID-19 period, indicating that patients residing in the hospital have a good adherence to their treatment, as they are monitored by the nurses in charge. A recent study showed that psychiatric patients who used antipsychotics and were adherent to their treatment were less likely to develop COVID-19 and had better outcomes following infection than the general population [20]. 

The regression analysis revealed that the interaction between being diagnosed with COVID-19 and cognitive function before the COVID-19 period was predictive of the cognitive function after the COVID-19 period. This association could be due to the immune-inflammatory response to the virus, which was not assessed in this study. Hence, it could be assumed that cognitive deficits might be due to SARS-CoV-2 infection rather than other factors such as the use of antipsychotics or the clinical symptoms of the disease. Evidence from studies suggests that immuno-inflammatory processes may play an essential role in cognitive impairments in different neuropsychiatric diseases [56]. Inflammation showed to be a paramount neuropathological process causing cognitive decline [57]. A meta-analysis showed that low-grade inflammation measured by an elevated blood level of C-reactive protein was a frequent denominator in the inflammatory cascade and has been linked to a high risk of schizophrenia [58]. Low-grade inflammation has also been associated with cognitive impairment, which is a common source of disability in people with schizophrenia [59]. The inflammatory cytokines, produced by the inflammation, bind to receptors on neurons, causing changes in mood, cognition, and behavior and leading to neurocognitive impairment in schizophrenia [60]. Future studies are necessary to define the range of cognitive impairments and the long-term effect of COVID-19 on cognition among patients with schizophrenia.

### Limitations

Our study has several limitations. Firstly, it was observational, and conclusions regarding causality could not be inferred. Secondly, the results could not be generalized to the whole schizophrenic population as the patients were selected from one site. Thirdly, the sample size was relatively small to identify any changes in cognitive domains. Also, the interval between the two assessments might be short for detecting any significant change for the variables tested. Information bias is also possible as two different evaluators collected the information and because some scales were self-reported; thus, accurate details could not be provided during the face-to-face interview. Medications patients were taking were recorded before and after the COVID-19 period, not during the disease, which could prevent detection of a direct effect of treatment on cognitive changes. Also, there was an imbalance between the number of patients with and without the SARS-CoV-2 infection. The reliability of the positive PANSS scale after the COVID-19 period was low. Some variables related to viral infections, such as the assessment of the inflammatory immune system or antiviral therapy, were not measured, which might affect the cognitive variation.

## Conclusion

Our study showed a deterioration in objective cognitive function among schizophrenic patients diagnosed with COVID-19. SARS-CoV-2 infection affected global cognition, verbal memory, and working memory, where patients diagnosed with COVID-19 had more deficits in these domains than those without COVID-19. The most predictive factor for cognition was the interaction between being diagnosed with COVID-19 and global cognition before the COVID-19 period. Depression and autonomy remained the same before and after the COVID-19 period. Our findings are preliminary; further studies are necessary to clarify the variation of cognitive function among schizophrenic patients with COVID-19 and the effect of antipsychotics, mainly chlorpromazine, on COVID-19 patients.

## Data Availability

The datasets used and/or analyzed during the current study are available from the corresponding author upon reasonable request.

## Abbreviations

COVID-19: Coronavirus disease of 2019
SARS-CoV-2: Severe acute respiratory syndrome coronavirus 2
WHO: World Health Organization
CNS: Central nervous system
HPC: Psychiatric Hospital of the Cross
RT-PCR: Real-time reverse transcriptase-polymerase-chain-reaction
DSM: Diagnostic and Statistical Manual of Mental Disorders
ADS: Anticholinergic Drug Scale
BACS: Brief Assessment of Cognition in Schizophrenia
PANSS: Positive and Negative Syndrome Scale
CDSS: Calgary Depression Scale for Schizophrenia
ADL: Activities of Daily Living
SPSS: Statistical Package for the Social Sciences
SD: Standard deviation
ARDS: Acute respiratory distress syndrome
MERS: Middle East respiratory syndrome coronavirus
